# First Report of *Joyeuxiella* spp. Infection in Eurasian Lynx and Its Histopathology Study from Iran: A Case Report

**Published:** 2020

**Authors:** Seyed Mohammad HOSSEINI, Amir Hossein MOSHREFI, Aryan ESFANDIYARI, Mohammad Reza YOUSSEFI, Alireza NASSIRI

**Affiliations:** 1. Department of Pathology, Babol Branch, Islamic Azad University, Babol, Iran; 2. Young Researchers and Elite Club, Babol Branch, Islamic Azad University, Babol, Iran; 3. Department of Veterinary Parasitology, Babol Branch, Islamic Azad University, Babol, Iran

**Keywords:** Eurasian lynx, *Lynx lynx*, *Joyeuxiella* spp., Histopathology, Iran

## Abstract

According to the last information of IUCN Red List, Eurasian lynx has been endangered since 2010. The population of this animal is heavily affected by infectious parasites. Two adult Eurasian lynx (one male and one female) were illegally hunted and found dead in Parvar National Park, Semnan Province, Iran. After the autopsy, the tissue and parasite samples were collected from gastrointestinal tract and transferred to 70% alcohol. Samples were recovered and identified as *Joyeuxiella* spp. Sonsino, 1889. Tissue samples were taken from the place of sticking of parasites on the intestinal wall, for histopathological examination, and were transferred to 10% buffered formalin. Following routine processes and H&E staining, the slides were examined microscopically. Main histopathological observations were epithelial hyperplasia and destruction, inflammatory cell infiltration in mucosa and submucosa of jejunum. To the best of the author’s knowledge, this is the first report and histopathological study of *Joyeuxiella* spp*.* in the world in Eurasian lynx.

## Introdution

The Eurasian lynx (*Lynx lynx*) is a medium- sized carnivorous cat from Felidae family. It has strong, partly long legs, with furred paws and short tail with a black tip, black stubbles of hair on its ears. (1). 80–130cm in head and body length, 11–24.5 cm in tail length and 60–75 cm in shoulder height makes the size of this animal twice as big as the Iberian lynx (*L. pardinus*) and the biggest among the genus lynx cats (2). Males and females are 18–30 kg and 8–21 kg in weighting respectively. It is twice as big as a Canadian lynx (*L. Canadensis*) (3). Generally, this animal is native to woodland area. It is introduced to Russia from the West of the Europe. In Iran, it is native to Alborz Mountain. There is more than 30 prey species with so many variable sizes in the Eurasian lynx’s diet, from mice to moose (4).

According to last information of the International Union for Conservation of Nature (IUCN) Red List, Eurasian lynx has been in “Endangered” (EN) condition since 2010. Parasite infection is common and most of the carnivorous mammals may have important effects on Eurasian lynx populations (5) especially in the young age of animals, as there are reports related to kitten death with high numbers of internal parasites in Scandinavia and Switzerland (5–7).

*Joyeuxiella Fuhrmann*, 1935 is a genus of Cestoda class. The adult Cestode inhabits in the small intestine of any carnivores ministering as definitive hosts (e.g. cat, dog, fox, wolf). Also, this tapeworm is commonly found in cats, however, the prevalence of *Joyeuxiella* spp. in foxes is higher than the other canids. It seems that, the dietary habits of foxes to ingest the intermediate hosts raise the possibility of *Joyeuxiella* spp. Although the first-intermediate host has not been determined, reptiles and small mammals serve as secondary intermediate hosts (8).

The infection of definitive host occurs by eating an infected intermediate or secondary intermediate host. The adult parasite sticks to the host’s mucosa of the intestine wall. The attachment applied by a rostellum, which carries over seven (usually 14 to 18) rows of thorn-like hooks (8, 9). The attachment of the helminth to the mucosa of intestine might cause marked injury affiliated with villous necrosis. However, in most cases, the infected animals do not show any clinical signs (8).

Although parasite infection is common in Eurasian lynx, there is no report of *Joyeuxiella* in this animal till now. With the author’s information, this is the first report and histopathologic study of infection with *Joyeuxiella* in Eurasian lynx (*L. lynx*) in the world.

## Case Report

In years 2015 and 2017, two adult *Eurasian lynxes* (one male and one female) were illegally hunted and found dead in Parvar National Park, Semnan Province, Iran (35°53’N to 36°10’N; 53°23’E to 53°48’E.). The male one weighed 14 kg. The female one weighed 12 kg.

Recording of gross lesions and the collecting of parasites from gastrointestinal tract were performed after necropsy. Also, tissue samples were taken from the parasite’s attachment places and samples fixed in 10% buffered formalin and following by routine proceedings and H&E staining, the slides evaluated microscopically. All the parasites were washed in a saline solution (NaCl 0.9%). Then, lactophenol solution was used for clearing the worms and the parasites identified by light microscope. Parasites measurement was performed using the TCapture software (Tucsen, Fuzhou, China).

**Fig. 1:**
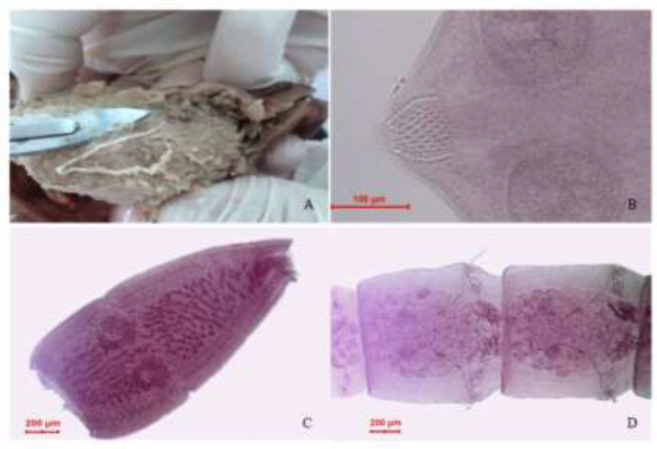
A: *Joyeuxiella* spp*.* on the intestine of *Lynx lynx*. B, C, D: Different parts of *Joyeuxiella* spp*.*

In the present study, intestine necropsy revealed that the Eurasian lynxes were infected by *Joyeuxiella* spp*.* The parasite was characterized by the morphology and the size of different part of body such as suckers, rostellum, and hooks. They were identified as the genus *Joyeuxeilla*, based on the morphology which comprised two sets of reproductive organs in a mature segment and in the gravid segments there was only one egg in each egg capsule (Fig.1) (9, 10). The length and width was 30–103mm, 0.71–2.45mm respectively, number of segment was 102–245, neck 190–1310 μm, maximum diameter of suckers (round or oval) was 121–153, rostellum size was 195–430 × 76–133 μm, mature segments size was 390–600 × 550–1100μm, the maximum diameter of ovary was 185–201μm, and the maximum diameter of egg size was 35–41μm.

Also the study of hooks showed 19–23 long near apex; 9–11 at base and base of rostellar invagination armed with irregularly arranged hooks. Microscopic examination showed epithelial hyperplasia and destruction, Goblet cell hyperplasia, inflammatory cell (Lymphocyte) infiltration in mucosa, and submucosa of jejunum the infected animals.

The study was approve by the Ethics Committee of Islamic Azad University, Babol, Iran.

## Discussion

Eurasian lynx (*L. lynx*) is a medium-sized cat that lives in the woodland area of Alborz Mountain in Iran. Hunting and vehicle collisions are the main unnatural causes of death and the main natural causes include starvation inter- and intraspecific killing and disease (11). In Switzerland, the death rates for the infectious diseases was 18% within which the amount of parasite infections was 53.85% (12). This shows that the infection with parasites may have important effects in this animal population (5). Thus, the information about parasite infection in this animal can be helpful.

Parasites identified in Eurasian lynx included *Diphyllobothrium latum*, *Spirometra janickii*, *Taenia pisiformis*, *T. laticollis*, *T. hydatigena*, *T. taeniaeformis*, *Trichinella* spp., Taeniidae, *Ancylostoma tubaeforme*, *Aelurostrongylus abstrusus*, *Alaria alata*, *Metastrongylus* spp., *Eucoleus aerophilus*, *Capilaria* spp., *Nematodirus* spp*.* (5, 6). To date, there has not been any report about occurrence of infection with *Joyeuxiella* spp*.* in Eurasian lynx in the world.

In Iran the prevalence of *Joyeuxiella* spp*.* in northern cities of Iran was 10%, and in Ilam city was 8.3% (13, 14). The study of intestinal parasite of stray dog in Lorestan Province of Iran showed that the prevalence of *Joyeuxiella* spp*.* was 5% (15). In Chaharmahal and Bakhtiari Province of Iran the parasite’s prevalence was 26.5%. In this study, same as previous reports, the prevalence rate of this parasite in foxes was higher than the other canids (16).The study of helminth parasites of stray dogs and jackals in Shahsavar area showed no infection with *Joyeuxiella* spp*.* (17).

According to previous studies, 65.8% of stray cats in Dubai, 58.3% in Baghdad, 55.2% in Mid-Ebro Valley of Spain, 38.3% in Kuwait, 31.8% in Egypt, 26% in Mosul were infected by this parasite (18–23). A study in Majorca Island of Spain about the rate of spread of helminth parasites in wild cats showed that the rate of infection with *Joyeuxiella* was high (76%).

Daoud et al reported *J. pasqualei* in cats in Iraq (24). Also, there have been some reports of infection with *J. echinorhyncoides* in jackals (South Africa), *Vulpes* sp*.*(Palestine), *Tatera indica* (Iran), fox (Kuwait, Iraq), and *Vulpes zerda* (USA) too (22).

The *J. echinorhyncoides* specimen, described by Jones in 1983, states the range of the length (21–145 mm), width (0.5–2.5 mm), number of segment (90–283), length of rostellum (188–329 × 56–80μm), maximum diameter of suckers (oval or round; 118–179 μm), length of neck (180–1620μm), size of Mature segments (380–694 × 523–1100 μm), number of hooks (17–26 long hooks near apex decreasing to 9–11 at base; base of rostellar invagination equipped with untidy arranged hooks.), maximum diameter of ovary (165–282 μm), egg size (35–45 μm) of this parasite. The range of the characterizing parameters in our study was in this range although the precise diagnosis is possible by PCR method. (22).

In Ilam Province of Iran, 10 species of internal parasite including helminthes, such as *J. echinorhyncoides* and protozoa were detected in wild cats (14). Histopathologic study of internal parasites showed that the infection with the endo parasite could affect all the organs in the body especially the small intestine and increased crypt length and mitotic rate of epithelial cells, inflammatory cell infiltration, vasodilatation, congestion, hemorrhage and necrosis with the destruction of the villi, degeneration in lamina propria of Intestinal villi (14). Our histopathologic study showed epithelial hyperplasia and destruction and inflammatory cell infiltration in mucosa and submucosa in jejunum of small intestine in both cases.

## Conclusion

Regarding to the author’s knowledge, this study might be the first case report and histopathological study of *Joyeuxiella* spp. isolated from Eurasian lynx in the world from central parts of Iran.
